# Clinical Characteristics, Complications and Outcomes of Patients with Severe Acute Respiratory Distress Syndrome Related to COVID-19 or Influenza Requiring Extracorporeal Membrane Oxygenation—A Retrospective Cohort Study

**DOI:** 10.3390/jcm10225440

**Published:** 2021-11-21

**Authors:** Kevin Roedl, Ahmel Kahn, Dominik Jarczak, Marlene Fischer, Olaf Boenisch, Geraldine de Heer, Christoph Burdelski, Daniel Frings, Barbara Sensen, Axel Nierhaus, Stephan Braune, Yalin Yildirim, Alexander Bernhardt, Hermann Reichenspurner, Stefan Kluge, Dominic Wichmann

**Affiliations:** 1 Department of Intensive Care Medicine, University Medical Centre Hamburg-Eppendorf, 20246 Hamburg, Germany; ahmel.kahn@stud.uke.uni-hamburg.de (A.K.); d.jarczak@uke.de (D.J.); mar.fischer@uke.de (M.F.); o.boenisch@uke.de (O.B.); deheer@uke.de (G.d.H.); c.burdelski@uke.de (C.B.); d.frings@uke.de (D.F.); b.sensen@uke.de (B.S.); nierhaus@uke.de (A.N.); s.kluge@uke.de (S.K.); d.wichmann@uke.de (D.W.); 2 Department of Medical Intensive Care and Emergency Medicine, St. Franziskus-Hospital, 48145 Münster, Germany; stephan.braune@sfh-muenster.de; 3 Department of Cardiovascular Surgery, University Heart and Vascular Center Hamburg, 20246 Hamburg, Germany; y.yildirim@uke.de (Y.Y.); al.bernhardt@uke.de (A.B.); reichenspurner@uke.de (H.R.)

**Keywords:** COVID-19, coronavirus disease, influenza, multiple organ failure, ARDS, ECMO, SARS-CoV-2

## Abstract

Extracorporeal membrane oxygenation (ECMO) represents a viable therapy option for patients with refractory acute respiratory distress syndrome (ARDS). Currently, veno-venous (vv) ECMO is frequently used in patients suffering from coronavirus disease 2019 (COVID-19). VV-ECMO was also frequently utilised during the influenza pandemic and experience with this complex and invasive treatment has increased worldwide since. However, data on comparison of clinical characteristics and outcome of patients with COVID-19 and influenza-related severe ARDS treated with vv-ECMO are scarce. This is a retrospective analysis of all consecutive patients treated with vv/(veno-arterial)va-ECMO between January 2009 and January 2021 at the University Medical Centre Hamburg-Eppendorf in Germany. All patients with confirmed COVID-19 or influenza were included. Patient characteristics, parameters related to ICU and vv/va-ECMO as well as clinical outcomes were compared. Mortality was assessed up to 90 days after vv/va-ECMO initiation. Overall, 113 patients were included, 52 (46%) with COVID-19 and 61 (54%) with influenza-related ARDS. Median age of patients with COVID-19 and influenza was 58 (IQR 53–64) and 52 (39–58) years (*p* < 0.001), 35% and 31% (*p* = 0.695) were female, respectively. Charlson Comorbidity Index was 3 (1–5) and 2 (0–5) points in the two groups (*p* = 0.309). Median SAPS II score pre-ECMO was 27 (24–36) vs. 32 (28–41) points (*p* = 0.009), and SOFA score was 13 (11–14) vs. 12 (8–15) points (*p* = 0.853), respectively. Median P/F ratio pre-ECMO was 64 (46–78) and 73 (56–104) (*p* = 0.089); pH was 7.20 (7.16–7.29) and 7.26 (7.18–7.33) (*p* = 0.166). Median days on vv/va-ECMO were 17 (7–27) and 11 (7–20) (*p* = 0.295), respectively. Seventy-one percent and sixty-nine percent had renal replacement therapy (*p* = 0.790). Ninety-four percent of patients with COVID-19 and seventy-seven percent with influenza experienced vv/va-ECMO-associated bleeding events (*p* = 0.004). Thirty-four percent and fifty-five percent were successfully weaned from ECMO (*p* = 0.025). Ninety-day mortality was 65% and 57% in patients with COVID-19 and influenza, respectively (*p* = 0.156). Median length of ICU stay was 24 (13–44) and 28 (16–14) days (*p* = 0.470), respectively. Despite similar disease severity, the use of vv/va-ECMO in ARDS related to COVID-19 and influenza resulted in similar outcomes at 90 days. A significant higher rate of bleeding complications and thrombosis was observed in patients with COVID-19.

## 1. Introduction

The severe acute respiratory syndrome coronavirus 2 (SARS-CoV-2) emerged in 2019 and caused an ongoing worldwide pandemic [1,2]. Although most infected patients have an asymptomatic or mild course of coronavirus disease 2019 (COVID-19) a considerable number of patients required hospitalisation [1,3,4]. About 5% of COVID-19 patients need treatment in the intensive care unit (ICU), mainly for respiratory support for varying degrees of ARDS as well as other forms of organ failure [1,5,6]. These patients have a high risk of mortality, especially when invasive mechanical ventilation (MV) is needed [6,7,8,9,10]. For patients who are experiencing progressive and refractory respiratory failure, veno-venous extracorporeal membrane oxygenation (vv-ECMO) may be considered as rescue therapy [11].

The use of vv-ECMO is an established rescue therapy in patients suffering from severe acute respiratory distress syndrome (ARDS) refractory to conservative management including optimised ventilator settings, prone positioning and pulmonary vasodilatory treatment [12]. Early initiation of vv-ECMO as well as early referral to ECMO centres has been proven to be beneficial in these patients [13,14,15].

Use of vv-ECMO in patients with ARDS related to viral infections was previously reported during the influenza A (H1N1) pandemic as well as the Middle East respiratory syndrome coronavirus (MERS-CoV) outbreaks [16,17]. Clinical features regarding clinical symptoms and course of disease of COVID-19 and influenza are highly variable, and recent studies described differences regarding patient characteristics and outcomes [18,19]. For the comparison of patients with severe ARDS related to COVID-19 and influenza requiring ECMO there is limited data regarding clinical characteristics, complications and outcomes [20,21,22,23].

In the present study, we aimed to investigate and compare clinical characteristics, outcomes and complications of patients with COVID-19 and influenza receiving ECMO for refractory severe ARDS in an experienced high-volume centre.

## 2. Materials and Methods

### 2.1. Study Population, Design and Ethics

We performed a retrospective analysis of all consecutive patients with COVID-19 or influenza admitted to the ICUs of the Department of Intensive Care Medicine at the University Medical Centre Hamburg-Eppendorf (Hamburg, Germany) between 1 January 2009 and 15 January 2021. The Department of Intensive Care Medicine cares for all critically ill adult patients of the university hospital and comprises 12 ICUs, with a total capacity of 140 beds. During the pandemic, a maximum of 3 ICUs with a total capacity of 36 beds was exclusively dedicated to the treatment of patients with COVID-19. The study was approved by the local clinical institutional review board and complies with the Declaration of Helsinki. The study was registered with the Ethics Committee of the Hamburg Chamber of Physicians (No.: WF-052/21). Owing to the retrospective character of the study and its pseudonymised data collection, the need for informed consent was waived.

### 2.2. Inclusion and Exclusion Criteria

We included all consecutive adult patients (≥18 years) with confirmed COVID-19 or influenza requiring vv- or veno-arterial (va)-ECMO support admitted to our department during the study period. Confirmed COVID-19 and influenza were defined as at least one positive result on reverse transcriptase polymerase chain reaction obtained from nasopharyngeal swabs and/or bronchial secretions. Patients with a non-completed ICU stay (ongoing ICU treatment at the end of the study period), without confirmed COVID-19/influenza or aged <18 years were excluded.

### 2.3. Data Collection

Data were collected from the department’s electronic patient data management system (PDMS, Integrated Care Manager^®^ (ICM), Version 9.1—Draeger Medical, Luebeck, Germany) and the department’s ECMO database. The extracted data included age, gender, comorbidities, admission diagnosis, length of ICU stay, treatment modalities and organ support (mechanical ventilation, prone positioning, type of ECMO (vv/va), vasopressor support and renal replacement therapy), blood products, medication such as glucocorticoid and anti-infective treatment, as well as laboratory test results.

### 2.4. Study Definitions and Patient Management

ARDS was defined according to the Berlin definition, using the PaO_2_/FiO_2_ ratio (Horowitz index) as marker for severity [24]. Severity of illness was evaluated by Sequential Organ Failure Assessment (SOFA) [25], Acute Physiology And Chronic Health Evaluation (APACHE II) [26] and Simplified Acute Physiology II (SAPS II) [27] score. Charlson Comorbidity Index (CCI) [28] was calculated for all patients. Clinical patient management was performed according to national and international guidelines, including prone positioning in moderate to severe ARDS and restrictive fluid management following the initial resuscitation period. Vasopressor support was initiated to obtain a mean arterial pressure (MAP) of 65 mmHg using norepinephrine [12,29]. Patients with severe hypoxemic and/or hypercapnic respiratory failure in combination with severe respiratory acidosis refractory to adjunctive therapies received vv-ECMO (CARDIOHELP-System Maquet GmbH, Rastatt, Germany; Novalung, Fresenius Medical Care, Bad Homburg, Germany; Stoeckert Centrifugal Pump Console, LivaNova, Munich, Germany). Criteria for the initiation of vv-ECMO support were based on the guidelines of the Extracorporeal Life Support Organization (ELSO) and national recommendations [29,30]. Prone positioning during ECMO therapy was initiated in patients with persistent severe hypoxemia. Presence of thrombosis was defined as pulmonary embolisms or deep vein thrombosis; the diagnosis was based on clinical suspicion and the subsequent confirmative diagnostic procedures (e.g., ultrasound or computed tomography) according to local standard operation procedures. Anticoagulation was performed using continuous application of unfractionated heparin. The effect of heparin was monitored using the activated clotting time. The targeted activated clotting time was 40 to 50 s in all patients. Patient survival was obtained at ICU discharge, after 28 and after 90 days post ECMO initiation.

### 2.5. Statistical Analysis

Data are presented as absolute numbers and relative frequency or median and with interquartile range (IQR). We performed an exploratory analysis; categorical variables were compared via chi-square analysis or Fisher’s exact test, as appropriate. Continuous variables were compared via Mann–Whitney U test. Survival function estimates were calculated using the Kaplan–Meier method and were compared by log rank test.

Statistical analysis was conducted using IBM SPSS Statistics Version 24.0 (IBM Corp., Armonk, NY, USA). A *p*-value of <0.05 was considered to be statistically significant. The study protocol was prepared in accordance with the Strengthening the Reporting of Observational Studies in Epidemiology recommendations.

## 3. Results

### 3.1. Study Population

**Figure 1:**
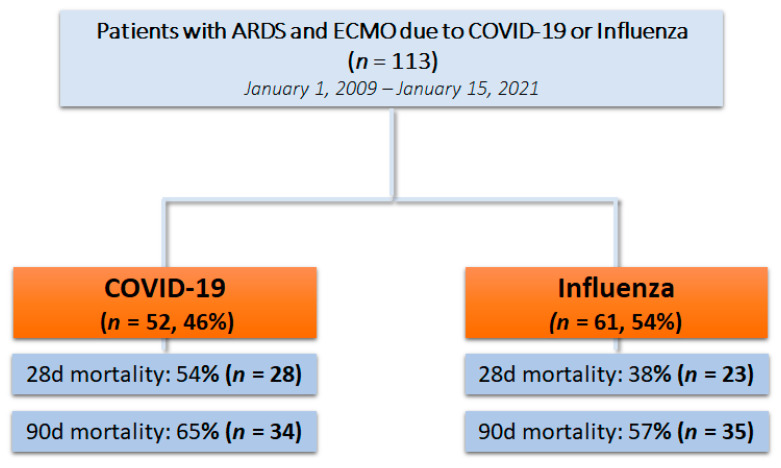
Study flow chart.

Overall, 113 patients were included who suffered from severe ARDS due to influenza pneumonia or COVID-19 and were treated with vv/va-ECMO during the study period. We could identify 61 (54%) patients with influenza-related disease (2009–2021) and 52 (46%) with COVID-19 (2020–2021). The study flow chart is shown in Figure 1.

### 3.2. Characteristics at Baseline

Detailed clinical characteristics on baseline and demographic characteristics are shown in Table 1. The median age was 52 (39–58) and 58 (53–64) years in patients with influenza and COVID-19, respectively (*p* < 0.001). Comorbidities displayed by CCI were median 2 (0–5) points in patients with influenza pneumonia and 3 (1–5) points in patients with COVID-19 (*p* = 0.309). Most common comorbidities were arterial hypertension (23% vs. 53%, *p* = 0.001), chronic lung disease (48% vs. 38%, *p* = 0.332), diabetes mellitus type II (15% vs. 38%, *p* = 0.004) and chronic kidney disease (15% vs. 2%, *p* = 0.002). Further comorbidities are displayed in Table 1 and Supplementary Table S1. The disease severities displayed by the median SAPS II, APACHE II and SOFA score on admission were 32 (28–41) vs. 27 (24–35.5) (*p* = 0.009), 17 (12–23) vs. 18 (14–22) (*p* = 0.885) and 10 (9–13) vs. 10 (8–13) (*p* = 0.877), respectively.

Overall, 75% (*n* = 46) and 90% (*n* = 47) of patients with influenza and COVID-19 were referred from other hospitals for vv/va-ECMO therapy (*p* = 0.038). On admission, the median paO_2_ was 55 (48–62) and 55 (48–62) mmHg (*p* = 0.514), and the pH was 7.25 (7.16–7.33) and 7.25 (7.16–7.33), respectively (*p* = 0.189). The median FiO_2_ on admission was 100 (85–100) and 100 (80–100) mmHg in the two groups, respectively. The corresponding paO_2_/FiO_2_ ratio on admission was 70 (55–81) and 69 (56–81), respectively (*p* = 0.607). Laboratory parameters before and after vv/va-ECMO therapy are displayed in Supplementary Table S2.

### 3.3. Patient Characteristics before ECMO Cannulation

Before vv/va-ECMO initiation, patients with influenza and COVID-19 presented a median paO_2_/FiO_2_ ratio of 73 (56–104) and 64 (46–78) (*p* = 0.087), the median paCO_2_ was 66 (55–84) and 75 (57–97) mmHg (*p* = 0.095) and the median pH was 7.26 (7.18–7.33) and 7.20 (7.16–7.29) (*p* = 0.166), respectively. The median respiratory rate on controlled mechanical ventilation was 30 (24–33) compared to 28 (26–30) per minute (*p* = 0.599). The median positive end expiratory pressure (PEEP) and inspiratory plateau pressure (pINSP) were 15 (12–17) and 32 (29–35) mmHg in patients with influenza and 14 (11–16) and 33 (30–35) in patients with COVID-19 (*p* = 0.176 and *p* = 0.289).

Therapies used as part of the algorithm for treatment of severe ARDS before vv/va-ECMO cannulation in patients with influenza and COVID-19 were prone positioning in 36% (*n* = 22) and 79% (*n* = 41) (*p* < 0.001), neuromuscular blockade in 25% (*n* = 15) and 58% (*n* = 30) (*p* < 0.001), inhaled vasodilatory treatment in 41% (*n* = 25) and 54% (*n* = 28) (*p* = 0.172) and glucocorticoid therapy in 26% (*n* = 16) and 71% (*n* = 37) (*p* < 0.001).

### 3.4. ECMO Specific Characteristics

The most common ECMO cannulation site in patients with influenza and COVID-19 was femoral/jugular in 77% (*n* = 47) and 96% (*n* = 50), followed by bilateral femoral in 21% (*n* = 13) and 2% (*n* = 1) of patients. Patients with influenza received vv-ECMO in 66% (*n* = 40) and va-ECMO in 34% (*n* = 21). For patients with COVID-19, 98% (*n* = 51) received vv-ECMO and 2% (*n* = 1) underwent va-ECMO therapy. Prone positioning during ECMO therapy was performed in 13% (*n* = 8) and 31% (*n* = 16) of patients with influenza and COVID-19, respectively (*p* = 0.022). A median of 1 (0–1) and 0 (0–1) membrane changes were performed during vv/va-ECMO therapy (*p* = 0.002). Further characteristics of vv/va-ECMO therapy are displayed in Table 2.

### 3.5. Further Organ Support

During vv/va-ECMO therapy, 96% (*n* = 108) of all patients received vasopressor support with norepinephrine, 93% with influenza and 98% with COVID-19 (*p* = 0.232). Renal replacement therapy was initiated in 69% (*n* = 42) and 71% (*n* = 37) of patients (*p* = 0.790), respectively. Fluid balance after start, 24 h and 7 d of vv/va-ECMO therapy was significantly higher in patients with influenza (*p* < 0.05); details are displayed in Table 2.

### 3.6. VV-/VA-ECMO Complications

The most common ECMO complication was bleeding, which occurred in 86% (*n* = 97) of all patients, 77% (*n* = 47) in influenza and 96% (*n* = 50) in patients with COVID-19 (*p* = 0.004). Clinically relevant thrombosis occurred in 13% (*n* = 8) and 40% (*n* = 21) of patients with influenza and COVID-19 (*p* = 0.004), respectively. We observed that 25 patients had deep vein thrombosis (influenza: 11% (*n* = 7), COVID-19: 35% (*n* = 18)) and 4 suffered from pulmonary embolism ((influenza: 2% (*n* = 1), COVID-19: 6% (*n* = 3)). Further details of complications are listed in Table 2. Overall, 17% (*n* = 19) of all patients experienced cardiac arrest during their ICU stay. Six percent (*n* = 7) occurred during vv/va-ECMO therapy, 3 patients of whom had influenza and 4 COVID-19 (*p* = 0.186).

### 3.7. Outcome

**Figure 2:**
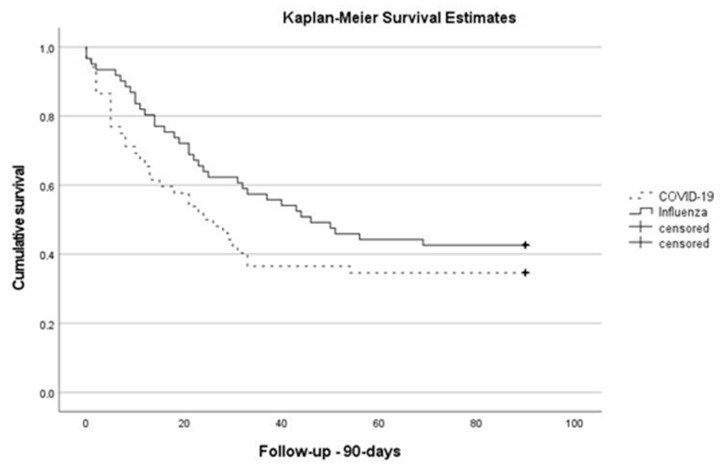
Kaplan–Meier 90-days survival estimates (log rank: *p* = 0.156).

Overall, in 58% of all patients, percutaneous tracheostomy was performed during their ICU stay, 59% (*n* = 36) in influenza and 58% (*n* = 30) in patients with COVID-19 (*p* = 0.887). The median length of vv/va-ECMO treatment in patients with influenza and COVID-19 was 11 (7–20) and 17 (7–27) days, respectively (*p* = 0.295). Fifty-six percent (*n* = 34) of patients with influenza and 35% (*n* = 18) with COVID-19 were successfully weaned off vv/va-ECMO. Eight patients in the influenza group and one patient in the COVID-19 group died after vv/va-ECMO weaning in the ICU. The 28-day mortality in the influenza group was 38% (*n* = 23) and 54% (*n* = 28) in patients with COVID-19 (*p* = 0.086), and the 90-day mortality was 57% (*n* = 35) and 65% (*n* = 34) (*p* = 0.384), respectively (see Figure 2). The median length of an ICU stay was 28 (16–44) and 24 (13–44) days (*p* = 0.470), respectively.

## 4. Discussion

In the present study we investigated the clinical characteristics and outcomes of critically ill patients with severe ARDS due to COVID-19 or influenza pneumonia on vv-/va-ECMO. To our knowledge this is the most comprehensive study comparing these two groups with severe viral pneumonia requiring vv/va-ECMO. Patients with COVID-19 experienced significantly higher rates of complications including thromboembolic events and bleeding on vv/va-ECMO. However, despite differences of baseline demographics and severity of illness, we found no significant differences in mortality up to 90 days after ECMO initiation.

Previous studies reported a higher rate of bleeding events in patients with COVID-19 on vv/va-ECMO compared to influenza [21,23]. In our cohort we observed more bleeding complications in COVID-19 than in influenza patients. This may be due to a more aggressive anticoagulation protocol owing to the higher rate of venous thromboembolism (VTE) reported in our cohort. Furthermore, many previous studies on patients with COVID-19 described a high rate of VTE especially in critically ill patients [31]. The pathophysiology linked to these observations is possibly linked to the COVID-19-associated substantial dysregulation of both inflammation and coagulation [31,32,33]. Previous studies have reported that biomarkers reflecting high systemic inflammation and coagulation activation are associated with worse outcomes [32]. In our cohort, patients with COVID-19 showed higher inflammatory activation during the whole course of disease than influenza patients, potentially reflecting higher severity of illness and risk of VTE. Surprisingly, D-dimer levels were lower than those in patients suffering from influenza. The contrasting higher rates of VTE in patients with COVID-19 may have been due to more severe endothelial injury [34]. However, there were no surrogate parameters of endothelial injury available in our retrospective analysis to further evaluate this hypothesis. To date, the optimal anticoagulation strategy in critically ill patients with COVID-19 on vv/va-ECMO is much debated, but remains unknown and warrants further investigation regarding both optimal coagulation testing as well as optimal and individualised dosing and timing of anticoagulation.

Another striking finding was that 17% of patients in our cohort suffered from cardiac arrest (CA) during their ICU stay. In general, CA occurring within the ICU is less frequent and usually affects only 2% of the general critically ill population [35,36]. Although not significantly different, a higher proportion of patients with COVID-19 suffered from CA in our cohort. This is in line with different recent reports of patients with COVID-19 [35,37,38]. In a previous study, we reported an incidence of 18% for cardiac arrest in critically ill COVID-19 patients with severe ARDS; about one-third of patients were on ECMO therapy [35]. In our cohort, we observed that most patients suffered CA prior to initiation of vv/va-ECMO but also a relevant number of patients suffered CA during therapy. Cause of CA during ECMO therapy was mainly hypoxia related (3 of 7), vagal related (2 of 7), pericardial tamponade (1 of 7) and myocardial infarction related (1 of 7). This further highlights the severity of illness in this vulnerable patient cohort. The occurrence of CA in patients with ARDS requiring vv/va-ECMO is unknown and its occurrence should be further investigated in future studies.

COVID-19 and seasonal influenza pneumonia are both viral respiratory infections with highly variable clinical presentation and a course ranging from asymptomatic cases to respiratory failure with varying degrees of ARDS [18,19,39]. Several studies investigated clinical differences of hospitalised patients with influenza and COVID-19. Most studies reported that patients with influenza were significantly younger and presented with a higher burden of comorbidities [18,19]. In contrast to these findings, we observed that patients with COVID-19 in our cohort presented with a higher number of comorbidities on admission compared to patients with influenza. This may be explained by the fact that patients with multiple comorbidities may be at higher risk for a more severe course of COVID-19 disease leading to both initiation of vv-ECMO and high mortality [1,3]. Furthermore, a more liberal use of vv-ECMO in recent years due to growing experience in our centre may have contributed to applying vv-ECMO in sicker patients [40]. However, high mortality rates were also reported in patients requiring ECMO during MERS-CoV outbreaks [16,41]. Furthermore, changes of clinical practice over the time course of the COVID-19 pandemic due to expanding clinical experience and evidence, especially with respect to anticoagulation, sedation and mechanical ventilation, may also have had an impact on outcome differences between the two study groups.

Tang et al. compared ARDS patients with influenza and COVID-19 and found that patients with COVID-19 had lower severity of illness on admission and lower SOFA score-adjusted mortality [39]. We can confirm the finding of lower severity of illness in patients with COVID-19 on admission. However, on the day of vv/va-ECMO initiation, severity of illness was comparable between both groups. Possibly a more rapid clinical deterioration of patients with COVID-19 after their initially more liberal ICU admission may explain the observed differences between the two groups regarding severity of illness on admission in our cohort.

Several studies investigated differences between COVID-19 and influenza in hospitalised patients. However, data comparing the clinical characteristics and outcome of these two groups with ARDS on vv-ECMO are scarce [20,21,22]. Reported mortality rates were mainly higher in patients with COVID-19 [20,21,22]. Clinical characteristics and differences between influenza and COVID-19 regarding severity of illness, demographic characteristics and age were comparable to our study [20,21,22]. In one study, vv-ECMO specific prognostic scores before initiation were lower than in our study, which may have been due to different ECMO entry criteria [21]. However, most previous studies included low numbers of patients with COVID-19, all treated in experienced centres and thus limiting the external validity. Furthermore, these studies evaluated patients from the early phases of the pandemic and changes in clinical practice over the course of the pandemic may further reduce generalisability. Additionally, overall experience with initiation and handling of vv-ECMO over the study period may also have had an influence. The largest study compared 53 patients with COVID-19 and 67 patients with influenza and did not show significant outcome differences [23]. Duration on vv/va-ECMO and ICU length of stay in this study was significantly longer in patients with COVID-19, which is in line with our findings. ICU treatment regarding vasopressor support, RRT and rate of tracheostomy was similar in both groups. Greater length of ICU stay for COVID-19 as opposed to influenza patients on vv/va-ECMO in that study may be explained by a higher burden of comorbidities and age.

Our study has several limitations. First, our study included a relatively small number of patients. Larger cohorts are needed to confirm our findings. Second, the data were derived from a single centre and were collected retrospectively. Third, our results in an experienced ECMO centre may not be transferable to other, less experienced, settings. Fourth, our study included patients from several waves of the COVID-19 pandemic. Changes in clinical practice over time may have influenced the outcome of critically ill patients with COVID-19 on vv-ECMO. Fifth, changes in practice and management over the study period from 2009 to 2021 may also have influenced outcomes and could also explain the difference observed in the use of prone position and neuromuscular blockade between influenza and COVID-19 group.

## 5. Conclusions

In conclusion, in our study population, 90-day outcomes of patients with severe ARDS on vv/va-ECMO were similar between COVID-19 and influenza patients despite differences in baseline demographic characteristics and comorbidities. Patients with COVID-19 on vv/va-ECMO had significantly higher complications, including thromboembolic and bleeding events, than patients with influenza on vv/va-ECMO. Further, larger studies are needed to confirm these preliminary results.

## Figures and Tables

**Table 1:** Baseline demographic and clinical characteristics on ICU admission.

Variables	All (*n* = 113)	Influenza (*n* = 61)	COVID-19 (*n* = 52)	*p*-Value
Age (years)	54 (46–60)	52 (39–58)	58 (53–64)	<0.001
Males	76 (67)	42 (69)	34 (65)	0.695
BMI (kg/m²)	30.5 (24.8–35.3)	26.3 (24.1–33.0)	32.1 (26.3–36.7)	0.021
Disease Severity				
SAPS II (pts.)	29 (25–38)	32 (28–41)	27 (24–35.5)	0.009
APACHE II (pts.)	17 (12–22)	17 (12–23)	18 (14–22)	0.885
SOFA (pts.)	10 (9–13)	10 (9–13)	10 (8–13)	0.877
Comorbidities				
Charlson comorb. index, pts.	3 (1–5)	2 (0–5)	3 (1–5)	0.309
Arterial hypertension (*n*, %)	42 (37)	14 (23)	28 (53)	0.001
Chronic kidney disease (*n*, %)	10 (9)	9 (15)	1 (2)	0.002
Coronary heart disease (*n*, %)	8 (7)	3 (5)	5 (10)	0.332
Congestive heart failure (*n*, %)	1 (1)	1 (2)	0 (0)	1.000
Diabetes mellitus (*n*, %)	29 (26)	9 (15)	20 (38)	0.004
Chronic lung disease (*n*, %)	49 (43)	29 (48)	20 (38)	0.332
Smoking (*n*, %)	23 (20)	15 (25)	8 (15)	0.226
Respiratory function—Admission				
paO2 (mmHg)	56 (48–63)	55 (48–62)	55 (48–62)	0.514
paO2/FiO2	69 (56–81)	70 (55–81)	69 (56–81)	0.607
paCO2 (mmHg)	61 (52–75)	60 (51–75)	60 (51–75)	0.757
pH (level)	7.27 (7.17–7.35)	7.25 (7.16–7.33)	7.25 (7.16–7.33)	0.189
FiO2 (%)	100 (80–100)	100 (85–100)	100 (80–100)	0.461
Respiratory rate (/min)	28 (22–32)	28 (22–32)	28 (22–31)	0.864
Tidal volume—min (ml)	276 (189–332)	251 (140–316)	287 (207–341)	0.078
PEEP—max (mmHg)	16 (12–18)	16 (15–20)	15 (12–16)	0.004
pINSP—max (mmHg)	31 (27–35)	32 (28–36)	30 (25–34)	0.086
Admission from				
Direct to ECMO Centre	20 (18)	15 (25)	5 (10)	0.038
Transfer from other hospital	93 (82)	46 (75)	47 (90)	0.038
Outcome				
Duration ICU stay (days)	26 (14–44)	28 (16–44)	24 (13–44)	0.470
ICU mortality	71 (63)	36 (59)	35 (67)	0.363

Data are expressed as n (%) or median (interquartile range); Abbreviations: kg, kilogram; m, metre; BMI, body mass index; pts, points; mg, milligram; SAPS, Simplified Acute Physiology Score; SOFA, Sequential Organ Failure Assessment; APACHE, Acute Physiology and Chronic Health Evaluation; ICU, intensive care unit.

**Table 2:** vv/va-ECMO specific characteristics.

Variables	All (*n* = 113)	Influenza (*n* = 61)	COVID-19 (*n* = 52)	*p*-Value
Disease Severity				
SOFA—before ECMO (pts.)	12 (9–15)	12 (8–15)	13 (11–14)	0.853
SOFA—day 7 ECMO (pts.)	9 (5–13)	11 (8–14)	6 (4–10)	<0.001
Scores Prior to Cannulation				
RESP—score (pts.)	−1 (−3–2)	1 (0–3)	−2 (−4–−1)	<0.001
PRESERVE ECMO—score (pts.)	4 (3–5)	4 (3–6)	5 (4–5)	0.354
ECMOnet—score (pts.)	5 (3–6)	5 (4–7)	5 (3–6)	0.284
ARDS algorithm therapies prior to cannulation, *n* (%)				
Prone positioning	63 (56)	22 (36)	41 (79)	<0.001
Neuromuscular blockade	45 (40)	15 (25)	30 (58)	<0.001
Inhaled vasodilator	53 (47)	25 (41)	28 (54)	0.172
Glucocorticoid therapy	53 (47)	16 (26)	37 (71)	<0.001
Therapies during ECMO, *n* (%)				
Prone positioning	24 (21)	8 (13)	16 (31)	0.022
Glucocorticoid therapy	69 (61)	28 (46)	41 (79)	<0.001
Neuromuscular blockade	73 (65)	34 (56)	39 (75)	0.033
Cannulation site, *n* (%)				0.012
Internal jugular	1 (1)	1 (2)	0 (0)	
Bilateral femoral	14 (12)	13 (21)	1 (2)	
Femoral and jugular	97 (86)	47 (77)	50 (96)	
Femoral and subclavian	1 (1)	0 (0)	1 (2)	
ECMO configuration, *n* (%)/median (IQR)				
Veno-venous, *n* (%)	91 (81)	40 (66)	51 (98)	<0.001
Veno-arterial, *n* (%)	22 (19)	21 (34)	1 (2)	<0.001
FiO2—24 h	100 (100–100)	100 (100–100)	100 (100–100)	0.721
FiO2—d7	100 (80–100)	100 (80–100)	100 (80–100)	0.687
Blood flow—24 h (l/min)	4.5 (3.8–5.1)	4.5 (3.5–5.2)	4.5 (4.0–5.0)	0.550
Blood flow—d7 (l/min)	4.2 (3.5–5.1)	4.2 (3.2–5.2)	4.2 (3.7–5.0)	0.578
Sweep-gas flow—24 h (l/min)	4.7 (3.5–6)	5.2 (4–7)	4 (3.5–5)	0.021
Sweep-gas flow—d7 (l/min)	4.5 (3–6.5)	5.8 (3.3–7)	4 (3–6.1)	0.371
Respiratory function–before ECMO start, median (IQR)				
paO2 (mmHg)	61.6 (47.1–75.1)	63.4 (53.8–83.0)	58.4 (45.1–71.2)	0.057
paCO2 (mmHg)	70.1 (55.2–91.2)	65.9 (54.6–83.7)	74.8 (56.9–96.8)	0.095
pH (level)	7.23 (7.16–7.31)	7.26 (7.18–7.33)	7.20 (7.16–7.29)	0.166
FiO2–respirator (%)	100 (97–100)	100 (95–100)	100 (100–100)	0.034
Respiratory rate (/min)	28 (25–32)	30 (24–33)	28 (26–30)	0.599
Tidal volume	371 (276–459)	361 (279–451)	382 (280–489)	0.677
PEEP (mmHg)	15 (11–16)	15 (12–17)	14 (11–16)	0.176
pINSP (mmHg)	32 (29–35)	32 (27–35)	33 (30–35)	0.289
paO2/FiO2	65 (49–80)	73 (56–104)	64 (46–78)	0.089
Procedures/Therapies, *n* (%)/median (IQR)				
Norepinephrine during ECMO	108 (96)	57 (93)	51 (98)	0.232
Dobutamine during ECMO	11 (10)	4 (7)	7 (13)	0.217
Epinephrine during ECMO	8 (7)	3 (5)	5 (10)	0.332
Renal replacement therapy	79 (70)	42 (69)	37 (71)	0.790
Fluid balance—before ECMO	2132 (358–6392)	1253 (220–5642)	4595 (1734–7152)	0.028
Fluid balance—first 24 h	2536 (1181–5496)	3005 (1455–6105)	2105 (828–3333)	0.029
Fluid balance—first 7 d	2169 (−41–7143)	3731 (1081–11062)	1030 (−490–3404)	0.005
Antibiotic therapy	105 (93)	55 (90)	50 (96)	0.216
Tracheostomy	66 (58)	36 (59)	30 (58)	0.887
Platelet transfusions	5 (2–9)	8 (3–11)	4 (2–7)	0.064
FFP units	14 (4–22)	15 (7–31)	6 (4–15)	0.190
RBC transfusions	13 (6–26)	14 (7–27)	12 (5–22)	0.328
Complications during ICU				
Membrane clotting	4 (4)	2 (3)	2 (4)	0.871
HIT II	11 (10)	3 (5)	8 (15)	0.061
DIC	6 (5)	3 (5)	3 (6)	0.841
Leg ischemia non-severe	5 (4)	4 (7)	1 (2)	0.232
Leg ischemia severe	5 (4)	4 (7)	1 (2)	0.232
Thrombosis	29 (26)	8 (13)	21 (40)	0.001
Bleeding	97 (86)	47 (77)	50 (96)	0.004
Membrane changes	1 (0–1)	1 (0–1)	0 (0–1)	0.002
Timing				
Length of ECMO	13 (7–23)	11 (7–20)	17 (7–27)	0.295
Length of ICU stay	26 (14–44)	28 (16–44)	24 (13–44)	0.470
Outcome				
28-day mortality	51 (45)	23 (38)	28 (54)	0.086
90-day mortality	69 (61)	35 (57)	34 (65)	0.384
ICU mortality	71 (63)	36 (59)	35 (67)	0.363
Weaning from ECMO	52 (46)	34 (56)	18 (35)	0.025
Death in ICU after weaning	9 (8)	8 (13)	1 (2)	0.103

Data are expressed as n (%) or median (interquartile range); Abbreviations: ICU, intensive care unit; ECMO, extracorporeal membrane oxygenation; pts, points; mg, milligram; FFP, fresh frozen plasma; RBC, red blood cell; DIC, disseminated intravascular coagulation; HIT, heparin-induced thrombocytopenia.

## Data Availability

Data sharing is not applicable to this article.

## Supplementary Materials

The following are available online at www.mdpi.com/article/10.3390/jcm10225440/s1, Supplementary Table S1: Pre-existing comorbidities stratified according to presence of influenza and COVID-19; Supplementary Table S2: Biomarkers stratified according to presence of influenza and COVID-19.
