# Supplementary material for: Clinical Characteristics, Complications and Outcomes of Patients with Severe Acute Respiratory Distress Syndrome Related to COVID-19 or Influenza Requiring Extracorporeal Membrane Oxygenation—A Retrospective Cohort Study
Source: J Clin Med. 2021 Nov 21;10(22):5440. doi: 10.3390/jcm10225440 (PMC8619058; doi:10.3390/jcm10225440)
Supplement: Supplementary file 1 [file jcm-10-05440-s001.zip › jcm-1417470-supplementary.pdf]

Supplementary Tables:

- **Supplementary Table 1** – Pre-existing comorbidities stratified according presence of influenza and COVID-19
- **Supplementary Table 1 -** Biomarkers stratified according presence of influenza and COVID-19

-

Supplementary Table S1 – Pre-existing comorbidities stratified according presence of influenza and COVID-19

Parameters	***All*** *(n = 113)*	***Influenza*** *(n = 61)*	***COVID-19*** *(n = 52)*	*p*-value
**AIDS** *n (%)*	1 (1)	1 (2)	0 (0)	0.354
**Cerebral arterial disease** *n (%)*	6 (5)	2 (3)	4 (8)	0.297
**Chronic lung disease** *n (%)*	49 (43)	29 (48)	20 (38)	0.332
**Chronic kidney disease** *n (%)*	10 (9)	9 (15)	1 (2)	**0.002**
**Congestive heart disease** *n (%)*	1 (1)	1 (2)	0 (0)	1.000
**Connective tissue disease** *n (%)*	9 (8)	3 (5)	6 (12)	0.195
**Coronary heart disease** *n (%)*	8 (7)	3 (5)	5 (10)	0.332
**Dementia** *n (%)*	0 (0)	0 (0)	0 (0)	1.000
**Diabetes Mellitus** *n (%)*	29 (26)	9 (15)	20 (38)	**0.004**
**Liver cirrhosis** *n (%)*	5 (4)	5 (8)	5 (10)	**0.035**
**Peripheral arterial disease** *n (%)*	0 (0)	0 (0)	0 (0)	1.000
**Malignancy (Tumor)** *n (%)*				
Solid tumor	1 (1)	1 (2)	0 (0)	0.354
Leukemia	6 (5)	3 (5)	3 (6)	0.841
Lymphoma	4 (4)	3 (5)	1 (2)	0.390
Solid tumor with metastases	0 (0)	0 (0)	0 (0)	1.000

Abbreviations: AIDS, acquired immune deficiency syndrome; *n*, number;

Supplementary Table S2 – Biomarkers stratified according presence of influenza and COVID-19

Variables	***All*** *(n = 113)*	***Influenza*** *(n = 61)*	***COVID-19*** *(n = 52)*	*p*-value
**Laboratory results**				
Haemoglobin – before ECMO	9.8 (8.6 – 12.4)	10.3 (8.7 – 12.0)	9.7 (8.1 – 12.4)	0.374
Haemoglobin – 24h ECMO	9.1 (8.0 – 10)	9.2 (8.3 – 10.3)	8.8 (7.8 – 9.9)	0.195
Haemoglobin – d7 ECMO	9.4 (8.6 – 10)	9.6 (8.9 – 10.5)	8.9 (8.3 – 9.6)	**0.005**
Bilirubin – before ECMO	1.1 (0.7 – 1.8)	1.0 (0.7 – 1.5)	1.3 (0.7 – 1.9)	0.359
Bilirubin – 24h ECMO	1.5 (1.0 – 2.2)	1.4 (0.9 – 2.4)	1.7 (1.1 – 2.2)	0.406
Bilirubin – d7 ECMO	1.5 (0.9 – 5.2)	1.7 (1.0 – 5.2)	1.5 (0.8 – 4.4)	0.525
Leukocytes – before ECMO	13.6 (8.4 – 19.0)	12.5 (8.0 – 18.7)	14.5 (10.5 – 19.7)	0.293
Leukocytes – min ECMO	7.1 (4.6 – 10.1)	7.0 (4.3 – 8.7)	7.7 (5.1 – 11.8)	0.151
Leukocytes – max ECMO	20.4 (15.5 – 27.7)	20.4 (15.5 – 29.1)	19.9 (15.4 – 26.4)	0.413
Thrombocytes – before ECMO	228 (144 – 338)	228 (144 – 338)	304 (203 – 386)	**0.002**
Thrombocytes – 24h ECMO	172 (107 – 268)	172 (107 – 268)	224 (150 – 279)	**0.005**
Thrombocytes – d7 ECMO	104 (75 – 167)	104 (75 – 167)	96 (80 – 137)	0.496
Quick – before ECMO	93 (74 – 101)	93 (74 – 106)	95 (80 – 107)	0.258
Quick – 24h ECMO	79 (63 – 96)	79 (63 – 96)	81 (66 – 100)	0.296
Quick – d7 ECMO	83 (66 – 101)	83 (66 – 101)	88 (66 – 102)	0.367
INR – before ECMO	1.06 (1.0 – 1.2)	1.1 (1.0 – 1.2)	1.0 (1.0 – 1.1)	0.059
INR – 24h ECMO	1.1 (1.02 – 1.26)	1.2 (1.0 – 1.3)	1.1 (1.0 – 1.2)	0.224
INR – d7 ECMO	1.1 (1 – 1.2)	1.1 (1.0 – 1.2)	1.1 (1.0 – 1.2)	0.364
Fibrinogen – before ECMO	5.3 (4.0 – 7.1)	5.3 (4.0 – 7.7)	5.3 (4.3 – 6.4)	0.603
Fibrinogen – 24h ECMO	4.8 (3.7 – 5.7)	4.7 (4.0 – 6.5)	4.8 (3.7 – 5.4)	0.409
Fibrinogen – d7 ECMO	4.0 (3.7 – 4.8)	4.0 (3.6 – 4.1)	4.1 (3.7 – 5.1)	0.483
D-Dimere – before ECMO	4.5 (2.9 – 11.0)	6.5 (3.1 – 19.4)	4.2 (2.9 – 9.5)	0.375
D-Dimere – 24h ECMO	7.0 (3.3 – 13.6)	8.7 (4.4 – 17.9)	5.5 (3.0 – 10.6)	**0.027**
D-Dimere – d7 ECMO	11.6 (6.8 – 23.8)	32.9 (14.2 – 34.0)	31.1 (14.7 – 35.2)	0.731
Creatinine – before ECMO	1.4 (0.8 – 2.0)	1.4 (0.8 – 2.0)	1.2 (0.7 – 2.0)	0.310
Creatinine – 24h ECMO	1.3 (0.8 – 2.1)	1.5 (1.0 – 2.2)	1.1 (0.7 – 1.8)	**0.033**
Creatinine – d7 ECMO	1.1 (0.7 – 1.8)	1.2 (0.7 – 2.1)	1.0 (0.8 – 1.5)	0.267
AST – before ECMO	91 (45 – 152)	110 (44 – 207)	80 (51 – 120)	0.153
AST – 24h ECMO	106 (54 – 229)	130 (62 – 215)	78 (42 – 237)	0.321
AST – d7 ECMO	73 (50 – 159)	77 (49 – 150)	71 (52 – 163)	0.961
ALT – before ECMO	44 (30 – 72)	44 (29 – 72)	45 (31 – 69)	0.922
ALT – 24h ECMO	47 (27 – 68)	47 (28 – 64)	45 (25 – 73)	0.990
ALT – d7 ECMO	42 (28 – 82)	42 (28 – 88)	43 (29 – 63)	0.810
gGT – before ECMO	110 (60 – 193)	92 (58 – 188)	113 (51 – 192)	0.537
gGT – 24h ECMO	85 (42 – 141)	72 (36 – 127)	113 (70 – 204)	0.315
gGT – d7 ECMO	175 (134 – 400)	206 (142 – 437)	116 (63 – 145)	0.437
CRP – before ECMO	239 (142 – 295)	227 (105 – 273)	248 (164 – 300)	0.082
CRP – 24h ECMO	215 (142 – 286)	208 (130 – 302)	221 (158 – 280)	0.824
CRP – d7 ECMO	141 (73 – 223)	138 (75 – 213)	148 (69 – 230)	0.641
LDH – before ECMO	558 (424 – 714)	613 (420 – 1085)	556 (449 – 637)	0.284
LDH – 24h ECMO	596 (445 – 986)	631 (459 – 1079)	561 (419 – 862)	0.276
LDH – d7 ECMO	552 (427 – 778)	567 (403 – 800)	536 (454 – 708)	0.910
PCT – before ECMO	1.3 (0.6 – 4.4)	2.5 (0.8 – 11.5)	1.1 (0.4 – 3.0)	**0.031**
PCT– 24h ECMO	2.4 (0.7 – 8.4)	4.4 (1.2 – 25.5)	2.2 (0.6 – 5.1)	**0.043**
PCT – d7 ECMO	1.1 (0.5 – 3.2)	2.1 (0.9 – 5.3)	0.9 (0.4 – 2.6)	0.130

Data are expressed as n (%) or median (interquartile range)

Abbreviations: PCT, procalcitonin; CRP, c-reactive protein; INR, international normalized ratio; AST, aspartate amino transferase; ALT alanine aminotransferase; gGT, gamma glutamyl transferase; LDH, lactate dehydrogenase;
